# Comparing effects of continuous glucose monitoring systems (CGMs) and self-monitoring of blood glucose (SMBG) amongst adults with type 2 diabetes mellitus: a systematic review protocol

**DOI:** 10.1186/s13643-020-01386-7

**Published:** 2020-05-31

**Authors:** Mingyue Zheng, Yunting Luo, Wei Lin, Adeel Khoja, Qian He, Shenqiao Yang, Xuan Zhao, Peng Hu

**Affiliations:** 1 Adelaide Medical School, University of Adelaide, Adelaide, 5005 Australia; 2 School of Health and Rehabilitation, Chengdu University of Traditional Chinese Medicine, Chengdu, 611137 China; 3 Center of Infectious Diseases, West China Hospital, Sichuan University, Chengdu, 610041 China; 4 Institute for Disaster Management and Reconstruction, Sichuan University, Chengdu, 610207 China; 5 School of Management, Chengdu University of Traditional Chinese Medicine, Chengdu, 611137 China; 6 School of Nursing, Chengdu University of Traditional Chinese Medicine, Chengdu, 611137 China; 7 School of Pharmacy, Chengdu University of Traditional Chinese Medicine, Chengdu, 611137 China

**Keywords:** Type 2 diabetes, Continuous glucose monitoring, Self-monitoring of blood glucose, Health technology assessment, Diabetes management, Systematic review

## Abstract

**Background:**

Continuous glucose monitorings (CGMs) have been used to manage diabetes with reasonable glucose control amongst patients with type 2 diabetes (T2D) in recent decades. CGMs measure interstitial fluid glucose levels to provide information about glucose levels, which identify fluctuation that would not have been identified with conventional self-monitoring. Self-monitoring of blood glucose (SMBG) is a classical tool to measure glycaemic changes. However, the effectiveness of glucose control, hypoglycemia, weight change, quality of life and user satisfaction, are needed to evaluate and compare CGMs and SMBG amongst adults with T2D.

**Methods:**

The review will compare the various forms of CGM systems (i.e flash CGM, real-time CGM, retrospective CGM) versus SMBG or usual intervention regarding diabetes management amongst adults with T2D. The following databases will be searched: Cochrane Library, PubMed, EMBASE, CINAHL, PsycINFO, Scopus and grey literature (ClinicalTrials.gov, PsycEXTRA, ProQuest Dissertations, Google Scholar and Theses Global) for the identification of studies. The studies involving adults (aged ≥ 18 years old) will be included. We will only include and summarise randomised clinical trials (RCTs) with respect to authors, publication type, year, status and type of devices. Studies published in English between February 2010 and March 2020, will be included as the field of CGMs amongst T2D patients has emerged over the last decade. Primary outcomes will be HbA1c (glycosylated haemoglobin level) (mmol/L), body weight (kg), time spent with hypoglycaemia (< 70 mg/dl) or hyperglycaemia (≥ 180 mg/dl), blood pressure (< 140/90 mmHg is considered as good management) and quality of life (understanding and feeling of living situation based on culture and value system). Secondary outcome measures will be user satisfaction (patient or treatment/intervention satisfaction or satisfaction scale) and barriers (physical and mental difficulties or issues). Study selection, data extraction and risk of bias assessment will be conducted independently by at least two reviewers. A third reviewer will determine and resolve discrepancies. Moreover, the quality of the evidence of the review will be assessed according to the Grading of Recommendations Assessment, Development and Evaluation tool (GRADE).

**Discussion:**

The review will synthesise evidence on the comparison between using CGMs and SMBG. The results will support researchers and health professionals to determine the most effective methods/technologies in the overall diabetes management.

**Systematic review registration:**

PROSPERO CRD42020149212

## Background

Type 2 diabetes (T2D) is the most prevalent form of diabetes mellitus, characterised by β cell dysfunction and insulin resistance. T2D is also a common global health problem and leads to severe damage and complications to the heart, kidneys, nerves, blood vessels and eyes over time [1]. By 2040, the number of diabetic patients (aged 20-79) worldwide is expected to increase to 642 million [2]. The effects of diabetes extend beyond the individual to affect their families and societies such as reducing employment and early retirement and thus increasing the economic burden [2].

Sustaining reasonable blood glucose control is important to manage T2D and avoid the short-and long-term diabetic complications including hypoglycaemia and vascular diseases [3, 4]. However, blood glucose levels can undergo large fluctuations after meals, secondary to daily activities and after sleep, consequently creating control difficulties [5]. The monitoring tools needed to achieve reasonable glycaemic control continue to evolve, including more convenient self-monitoring blood glucose (SMBG) metres, continuous glucose monitorings (CGMs), and a better understanding of the strengths and limitations of glucose measurement. SMBG and CGMs are the most common tools for blood glucose monitoring amongst patients with T2D. SMBG is a traditional method for glucose measurement [4], which requires a manual finger-prick blood test and checking the blood glucose levels with a glucometre. SMBG is inconvenient to afford a complete set of the full profile of blood glucose fluctuation. By contrast, CGMs can provide more comprehensive profile of blood glucose levels of individuals [6]. Overall, CGMs have been generally accepted by patients with T2D, which also play an essential role in diabetes management.

Clinical application of CGMs started in the year 2000 and was generally indicated as a significant improvement in diabetes management [7]. Over the last decade, CGMs have been demonstrated to be clinically valuable due to its accuracy, convenience and improvement of software [8]. CGMs can promote glycaemic and weight control, to help reduce the risk of hypoglycaemia and hyperglycaemia, and improve relevant lifestyle behaviours [7–9]. It has become a useful tool for real-time monitoring of blood glucose in clinical and public diabetes management settings, and for assessing the impact of treatment and lifestyle on daily changes in blood glucose levels [8, 10]. CGM devices are intravascular devices, which can be minimally invasive, or even non-invasive. There are different types of CGM devices that can be defined in clinical practice: retrospective systems, real-time systems, and flash or intermittently viewed system et al. [6, 11]. A reduced requirement for frequent calibration has accompanied the improvement in accuracy of CGM sensors. For example, flash CGM could be considered a unique subset of CGMs which forms the blood glucose values only when the user scans the sensor by passing a cell phone or a reader near the sensor instead of updating a show of blood glucose continuously every five min intervals [7]. However, several potential common patient-reported barriers of CGM use including sensor insertion with pain or problems of high costs, accidental removal of the device or the adhesive strip and skin reactions of sensor adhesion [12, 13].

A review published in 2009 analysed the CGM data by statistical tools and then strongly encouraged researchers and clinicians to adopt the rich information contained in CGM data to guide their research and clinical practice, the CGM reports can provide the feedback and suggestion to diabetic patients [14]. A meta-analysis of four randomised clinical trials (RCTs) of a systematic review indicated that real-time CGM has better control in reducing HbA1c levels compared with SMBG amongst patients with T2D [4], even those RCTs in this review had inadequate sample size for achieving the legacy impacts. Besides, many of the included studies of the systematic review were short-term with a small number of participants. Another systematic review indicated that real-time CGM and professional CGM compared with usual care are effective in improving HbA1c control but could not form the conclusions on the effectiveness of flash CGM due to insufficient evidence [15]. A recent meta-analysis published in 2019 found that CGM can reduce HbA1c levels and time spent with hypoglycaemia amongst T2D. However, it has only searched three databases so that it may not include all relevant studies. Besides, it did not compare different types of CGM devices [16].

There is uncertainty between different CGM interventions/systems (i.e. real-time system, flash or intermittently reviewed system, retrospective system) and outcomes such as hypoglycaemia, weight change, quality of life and user satisfaction. A systematic review is also lacking about comparing those aspects between CGM systems and SMBG. Therefore, this systematic review of RCTs aims to evaluate the effects of CGMs vs SMBG on blood glucose levels, body weight, blood pressure, hypoglycaemia, quality of life and user satisfaction amongst adults with T2D.

## Methods

This protocol follows the guidelines of the Preferred Reporting Items for Systematic Review and Meta-Analysis Protocols (PRISMA-P) [17] (Additional file 1). This protocol will guide the further review and any deviations while conducting the review will be reported including the reasons for the changes made in the method section of the final published manuscript. The review has been registered in the International Prospective Register of Systematic Reviews (PROSPERO) CRD42020149212.

### Eligibility criteria

The inclusion criteria of the review are (a) study design as having RCTs only; (b) studies conducted in adults (aged ≥ 18 years old) diagnosed with T2D; (c) have defined any type of CGMs as the intervention group and SMBG or routine glucose monitoring as the control group and (d) studies published in English. Studies published between February 2010 and March 2020 will be included as the field of CGMs amongst T2D has emerged over the last decade. Abstracts will be eligible for inclusion if sufficient information is provided to judge the quality and potential for bias of these trials. Non-RCT studies, non-T2D, follow-up duration less than 6 weeks, conference abstracts and duplicate studies will be excluded.

Studies involving adults amongst T2D (aged ≥ 18 years old) have adopted CGMs as interventions will be included in this review. Inclusion criteria: (a) adults diagnosed with T2D (diagnostic criteria including the American Diabetes Association, World Health Organization or national guidelines) and (b) T2D of at least 8 weeks duration. Exclusion criteria: (a) adolescents (aged < 18 years old); (b) other types of diabetes (i.e. gestational diabetes mellitus or idiopathic diabetes or type 1 diabetes) and (c) patients in hospitalisation or intensive care unit or with serious diseases.

### Information sources and search strategy

The following databases will be searched: Cochrane Library, PubMed, EMBASE, CINAHL, PsycINFO, Scopus and grey literature (ClinicalTrials.gov, PsycEXTRA, ProQuest Dissertations, Google Scholar and Theses Global) for the identification of studies. The studies involving adults (aged ≥ 18 years old) will be included. We will include and summarise RCTs with respect to authors, publication type, year, status, and type of devices et al. Besides, the inclusion of grey literature (i.e. non-published, internal or non-reviewed articles, repositories) in the systematic review may help to overcome the publication bias that may arise due to the selective availability of data [18], thereby this review will include grey literature after reviewing the title and abstract accordingly.

Additionally, the reference list of identified systematic reviews and RCTs will also be updated to identify if references or bibliographies include relevant studies that might be included for the review (cross-referencing). Furthermore, indexed keywords in the Medical Subject Headings will be used to guarantee unified search terms. A comprehensive PubMed search strategy (Additional file 2) was developed in consultation with a medical librarian experienced in systematic database searching. Other databases will be searched and corresponding search strategies and logic grid will be adopted (Additional file 3).

### Study selection

Citation management system (Endnote X9) will be adopted to manage records exported from all the databases. First, all the studies will be screened by their titles and abstracts through an online software known as Covidence (https://www.covidence.org). Then, the full text of nominated studies will be screened according to the pre-defined eligibility criteria. Study selection, data extraction and risk of bias assessment will be conducted independently by at least two reviewers (MZ and YL). Both reviewers will describe outcome measures after reviewing the studies to confirm the relevance of RCTs. A third author (WL) will determine and resolve discrepancies to make the final decision about whether the study meets the eligibility criteria for being included in the review in a consensus meeting. The process of study screening and selection will be reported as the PRISMA flow diagram [19] (Additional file 4).

### Data extraction and management

A data extraction form (Additional file 5) was designed based on the suggestion of data extraction and synthesis by the Joanna Briggs Institute [20]. It covered the information of population, intervention, comparison intervention and outcome measures. To ensure the reliability of the data extraction process, it was designed and edited by the review team after consultation and was pilot tested by two independent reviewers (MZ and TY). To be specific, we will extract data by the form about characteristics of the studies to be included in the current study (including author, publication year, country, sample size, types of CGM devices, duration of diabetes, patient’s baseline, clinic history, basic treatment and intervention/treatment duration). Primary outcomes will be HbA1c (glycosylated haemoglobin level) (mmol/L), body weight (kg), time spent with hypoglycaemia (< 70 mg/dl) or hyperglycaemia (≥ 180 mg/dl), blood pressure (< 140/90 mmHg considers good management) and quality of life (understanding of the living situation based on culture and value system [21]). Secondary outcome measures will be user satisfaction (patient and/or treatment/intervention satisfaction on a satisfaction scale) and barriers (physical and mental difficulties or issues). Continuous variables will be demonstrated as mean values, standard deviations (SD), standard errors, or 95% confidence interval (CI) as and where applicable, whereas binary variables will be expressed as frequencies and percentages (%). Studies comparing one SMBG group with two or more intervention groups will be treated as two or more studies sharing an SMBG group. Two reviewers (MZ and YL) will independently evaluate the quality of each study that meet the inclusion criteria of the systematic review. Another researcher (WL) will provide judgement when two authors have different reviews if necessary. We will evaluate duplicate publications, assess all available data simultaneously, maximising the extraction of data for a bias assessment precisely. Authors will be contacted by emails to acquire missing or relevant material of their publications if necessary.

### Risk of bias of included studies

The latest revised Cochrane’s risk of bias tool will be used for evaluating the quality of randomised trials [22]. The following seven constructs will be evaluated as low, moderate and high risk of bias or unclear risk of bias: random sequence generation, allocation concealment, blinding of personnel and participants, blinding of outcome assessors, incomplete data, selective reporting and other potential risks (Additional file 6).

### Strength of evidence

According to the Grading of Recommendations Assessment, Development and Evaluation tool (GRADE) [23], the strength of evidence of the review will be assessed from the following parts; risk of bias, inconsistency, indirectness, imprecision, publication bias, other factors and upgrading. Two reviewers (MZ and YL) will independently evaluate the quality of each study. Any disagreement at this stage will be discussed and resolved by consultation and consensus of a third reviewer (WL). The summary of the quality of all included studies will be presented in a table which will follow the principle of GRADE.

### Data synthesis and statistical analysis

This systematic review will also synthesise a quantitative analysis known as meta-analysis. It seems that the quantitative units may express the continuous variables differently in each included study; analysis will be performed using standardised mean differences (SMD) or mean differences (MD) with its respective 95% CIs. Binary variables will be analysed and reported using risk ratio (RR) or odds ratio (OR) with its respective 95% CIs.

The sensitivity analysis will be used to test the robustness of the choices made, such as changing the cut-off for high- or low-quality included studies. Besides, the heterogeneity (deduced by the *I*^2^ statistics) will be described via reporting differences in the study design and the characteristics of the study population [24]. This systematic review intents to generalise the results beyond the included studies (generalisation inference). Besides, the patients of T2D frequently different in public health filed at baseline and duration of diabetes, which seem multiple study population of T2D [25]. Therefore, a random effect model will be used for analysis if appropriate, such as over five studies are included [26]. Be specific, *I*^2^ will be used for evaluating statistical heterogeneity (*I*^2^ ≥ 50% is considered as heterogeneous), since it is the percentage of total variation provided between the studies (*I*^2^ values of 75%, 50% and 25% represent high, moderate and low heterogeneity, respectively) [27].

If sufficient RCTs are available and variability amongst those studies is low, i.e. they are homogenous, a meta-analysis will be conducted as per the following subgroups if appropriate: (a) real-time CGM vs SMBG; (b) resorptive CGM vs SMBG; (c) real-time CGM vs routine care; (d) resorptive CGM vs routine care; and (e) real-time CGM vs resorptive CGM. According to these categories, measures of associations such as relative risks, and odds ratios will be synthesised and reported. In addition to this, when the number of included studies for this review is more than ten, a funnel plot will be plotted for evaluating publication bias. We will use the Eggers’ regression test to evaluate the asymmetry of the funnel plot statistically. Moreover, subgroup analysis via baseline HbA1c levels (< 6.5%, 6.6-7.9%, 8.0-11.0% and > 11.0%) will be performed to assess the impact of baseline HbA1c on the effectiveness of CGM. Additionally, if heterogeneity is identified, a meta-regression analysis will be conducted on whether baseline information such as HbA1c, gender, age and frequency and types of CGM sensor/device use has affected the impact of CGM on HbA1c levels.

## Discussion

The main aim of this review is to compare the effectiveness between GCMs and SMBG or routine care. A systematic review in this area is lacking to not only analyse the effectiveness of glucose control but also body weight, blood pressure, hypoglycaemia, quality of life and user satisfaction amongst adults with T2D. Moreover, it will also analyse the subgroups by comparing the differences between various types of CGM devices to help further in the improvement of developing these devices.

This review will have some advantages. The pre-defined approach is according to the Cochrane Handbook for Systematic Reviews of Interventions and considers the risk of bias and GRADE assessment [28, 29]. Intervention and control groups will be evaluated jointly and separately, so this systematic review will be able to determine why CGMs work as an intervention and under what circumstances. Therefore, risks of systematic error, random error and design error of the review will be avoided [30].

A potential limitation of this systematic review could be that the inclusion of grey literature without peer review might reduce the impact of the results. In addition to this, the review will only include RCTs since the comparison of the two interventions is only possible in that study design. The results of this review will be publicly available and will be disseminated via academic presentations (both locally and internationally) and through peer-reviewed publications. The review is anticipated to classify research gaps in the existing literature and provide evidence for further studies regarding CGM interventions and effective implementation.

## Supplementary information

**Additional file 1: ** Preferred Reporting Items for Systematic Review and Meta-Analysis Protocols (PRISMA-P) and checklist.

**Additional file 2: ** Preliminary search strategy for PubMed.

**Additional file 3: ** Logic grid.

**Additional file 4: ** PRISMA-2009 flow diagram.

**Additional file 5: ** Data extraction form.

**Additional file 6: ** Classification of randomised trials of biases.

## Data Availability

All data that will be analysed as a result of this study will be made available through a data sharing website, e.g. figshare or as additional files.

## Abbreviations

CGM: Continuous glucose monitoring
T2D: Type 2 diabetes
SMBG: Self-monitoring blood glucose metres
RCTs: Randomised controlled trials
HbA1c: Glycosylated haemoglobin
GRADE: Grading of Recommendations Assessment, Development and Evaluation tool
PROSPERO: International Prospective Register of Systematic Reviews
PRISMA: Preferred Reporting Items for Systematic Reviews and Meta-analyses
SD: Standard deviation
CI: Confidence interval
SMD: Standardised mean difference
MD: Mean difference
RR: Risk ratio
OR: Odd ratio

## References

[1] Bommer Christian, Sagalova Vera, Heesemann Esther, Manne-Goehler Jennifer, Atun Rifat, Bärnighausen Till, Davies Justine, Vollmer Sebastian (2018). Global Economic Burden of Diabetes in Adults: Projections From 2015 to 2030. Diabetes Care.

[2] Association AD. 2. Classification and diagnosis of diabetes: standards of medical care in diabetes-2019. Diabetes Care. 2019;42(Suppl 1):S13–28. 10.2337/dc19-S002 30559228

[3] Ajjan Ramzi A. (2017). How Can We Realize the Clinical Benefits of Continuous Glucose Monitoring?. Diabetes Technology & Therapeutics.

[4] Poolsup Nalinee, Suksomboon Naeti, Kyaw Aye (2013). Systematic review and meta-analysis of the effectiveness of continuous glucose monitoring (CGM) on glucose control in diabetes. Diabetology & Metabolic Syndrome.

[5] Ajjan Ramzi A, Cummings Michael H, Jennings Peter, Leelarathna Lalantha, Rayman Gerry, Wilmot Emma G (2018). Accuracy of flash glucose monitoring and continuous glucose monitoring technologies: Implications for clinical practice. Diabetes and Vascular Disease Research.

[6] Danne T, et al. International consensus on use of continuous glucose monitoring. Diabetes Care. 2017;40(12):1631–40. 10.2337/dc17-1600 PMC6467165 29162583

[7] Rodbard David (2017). Continuous Glucose Monitoring: A Review of Recent Studies Demonstrating Improved Glycemic Outcomes. Diabetes Technology & Therapeutics.

[8] McGill Janet B., Ahmann Andrew (2017). Continuous Glucose Monitoring with Multiple Daily Insulin Treatment: Outcome Studies. Diabetes Technology & Therapeutics.

[9] New J. P., Ajjan R., Pfeiffer A. F. H., Freckmann G. (2015). Continuous glucose monitoring in people with diabetes: the randomized controlled Glucose Level Awareness in Diabetes Study (GLADIS). Diabetic Medicine.

[10] Shibusawa Ryo, Yamada Eijiro, Okada Shuichi, Nakajima Yasuyo, Bastie Claire C., Yamada Masanobu (2018). The Impact of Short-Term Professional Continuous Glucose Monitoring on Glycemic Control Via Lifestyle Improvement. Diabetes Technology & Therapeutics.

[11] Adolfsson Peter, Parkin Christopher G, Thomas Andreas, Krinelke Lars G (2018). Selecting the Appropriate Continuous Glucose Monitoring System – a Practical Approach. European Endocrinology.

[12] Patton Susana R. (2015). Adherence to Glycemic Monitoring in Diabetes. Journal of Diabetes Science and Technology.

[13] Kovatchev B, et al. Comparison of the numerical and clinical accuracy of four continuous glucose monitors. Diabetes Care. 2008;31(6):1160–4. 10.2337/dc07-2401 PMC4607511 18339974

[14] Clarke W, Kovatchev B. Statistical tools to analyze continuous glucose. Diabetes Technol Ther. 2009;11(S1):S45–54. 10.1089/dia.2008.0138 PMC2903980 19469677

[15] Park Cindy, Le Quang A. (2018). The Effectiveness of Continuous Glucose Monitoring in Patients with Type 2 Diabetes: A Systematic Review of Literature and Meta-analysis. Diabetes Technology & Therapeutics.

[16] Ida S (2019). Utility of real-time and retrospective continuous glucose monitoring in patients with type 2 diabetes mellitus: a meta-analysis of randomized controlled trials. J Diabetes Res.

[17] Moher D (2015). Preferred reporting items for systematic review and meta-analysis protocols (PRISMA-P) 2015 statement. Syst Rev.

[18] Hopewell S (2007). Grey literature in meta-analyses of randomized trials of health care interventions. Cochrane Database Syst Rev.

[19] Moher D (2009). Preferred reporting items for systematic reviews and meta-analyses: the PRISMA statement. PLoS Med.

[20] Munn Z, et al. Data extraction and synthesis the steps following study selection in a systematic review. Am J Nurs. 2014;114(7):49–54. 10.1097/01.NAJ.0000451683.66447.89 25742352

[21] WHO. WHOQOL: measuring quality of life. Health statistics and information systems [cited 2020 4/10/2020 ].

[22] Sterne JAC (2019). RoB 2: a revised tool for assessing risk of bias in randomised trials. BMJ.

[23] Guyatt Gordon H, Oxman Andrew D, Vist Gunn E, Kunz Regina, Falck-Ytter Yngve, Alonso-Coello Pablo, Schünemann Holger J (2008). GRADE: an emerging consensus on rating quality of evidence and strength of recommendations. BMJ.

[24] Higgins Julian P. T., Thompson Simon G. (2002). Quantifying heterogeneity in a meta-analysis. Statistics in Medicine.

[25] Borenstein Michael, Hedges Larry V., Higgins Julian P.T., Rothstein Hannah R. (2010). A basic introduction to fixed-effect and random-effects models for meta-analysis. Research Synthesis Methods.

[26] Tufanaru Catalin, Munn Zachary, Stephenson Matthew, Aromataris Edoardo (2015). Fixed or random effects meta-analysis? Common methodological issues in systematic reviews of effectiveness. International Journal of Evidence-Based Healthcare.

[27] Higgins J. P T (2003). Measuring inconsistency in meta-analyses. BMJ.

[28] Higgins JP, et al. Cochrane handbook for systematic reviews of interventions version 6.0: Cochrane; 2019.

[29] Guyatt Gordon, Oxman Andrew D., Akl Elie A., Kunz Regina, Vist Gunn, Brozek Jan, Norris Susan, Falck-Ytter Yngve, Glasziou Paul, deBeer Hans (2011). GRADE guidelines: 1. Introduction—GRADE evidence profiles and summary of findings tables. Journal of Clinical Epidemiology.

[30] Keus F, et al. Evidence at a glance: error matrix approach for overviewing available evidence. BMC Med Res Methodol. 2010;10(1):90. 10.1186/1471-2288-10-90 PMC2959031 20920306

