# Supplementary material for: Comparing effects of continuous glucose monitoring systems (CGMs) and self-monitoring of blood glucose (SMBG) amongst adults with type 2 diabetes mellitus: a systematic review protocol
Source: Syst Rev. 2020 May 31;9:120. doi: 10.1186/s13643-020-01386-7 (PMC7262745; doi:10.1186/s13643-020-01386-7)
Supplement: Supplementary file 2 — Additional file 2: Preliminary search strategy for PubMed. [file 13643_2020_1386_MOESM2_ESM.docx]

**Additional file 2: Preliminary search strategy for PubMed**

***Comparing effects of continuous glucose monitoring systems (CGMs) and self monitoring of blood glucose (SMBG) among adults with type 2 diabetes mellitus: A systematic review protocol***

| **#** | **Searches** | Results |
| --- | --- | --- |
| 1 | ("Diabetes mellitus, type 2"[mh] OR Type 2 diabetes[tiab] OR Ketosis resistant diabetes mellitus[tiab] OR Non insulin dependent diabetes[tiab] OR Stable diabetes mellitus[tiab] OR Diabetes mellitus[tiab] OR NIDDM[tiab] OR Maturity onset diabetes mellitus[tiab] OR MODY[tiab] OR Slow onset diabetes mellitus[tiab] OR Noninsulin-dependent diabetes mellitus[tiab] OR Noninsulin dependent diabetes mellitus[tiab] OR Maturity onset diabetes[tiab] OR Adult-Onset Diabetes Mellitus[tiab]) | [316434](https://www-ncbi-nlm-nih-gov.proxy.library.adelaide.edu.au/pubmed/?cmd=HistorySearch&querykey=1) |
| 2 | (Continuous glucose monitoring system*[tiab] OR continuous glucose monitor*[tiab] OR continuous glucose sen*[tiab] OR continuous glucose device*[tiab] OR continuous blood sugar monitor*[tiab] OR continuous blood sugar sen*[tiab] OR continuous blood device*[tiab] OR continuous subcutaneous glucose monitor*[tiab] OR continuous subcutaneous glucose sen*[tiab] OR CGM*[tiab] OR real-time CGM*[tiab] OR rt-CGM*[tiab] OR flash glucose monitor*[tiab] OR FGM*[tiab] OR sensor-augmented insulin pump[tiab] OR SAP[tiab] OR iPro*[tiab] OR FreeStyle Libre*[tiab] OR HiBell*[tiab] OR Dexcom*[tiab] OR MiniMed*[tiab] OR Medtronic*[tiab] OR Guardian Connect CGM*[tiab] OR Senseonics Eversense*[tiab] OR GlucoTrack*[tiab]) | [51045](https://www-ncbi-nlm-nih-gov.proxy.library.adelaide.edu.au/pubmed/?cmd=HistorySearch&querykey=2) |
| 3 | ("blood glucose self-monitoring"[mh] OR Blood Glucose Self-Monitoring[tiab] OR Blood Sugar Self-Monitoring[tiab] OR Home Blood Glucose Monitoring[tiab] OR Blood glucose monitoring system*[tiab]) | 6947 |
| 4 | (randomized controlled trial[pt] OR controlled clinical trial[pt] OR randomized[tiab] OR placebo[tiab] OR “clinical trials as topic”[majr] OR randomly[tiab] OR trial[ti]) | [1184611](https://www-ncbi-nlm-nih-gov.proxy.library.adelaide.edu.au/pubmed/?cmd=HistorySearch&querykey=4) |
| 5 | 1 AND 2 AND 3 AND 4 | [12331](https://www.ncbi.nlm.nih.gov/pubmed/?cmd=HistorySearch&querykey=15) |
| 6 | English [Language] | [26139740](https://www-ncbi-nlm-nih-gov.proxy.library.adelaide.edu.au/pubmed/?cmd=HistorySearch&querykey=6) |
| 7 | Humans [Filter] | [18397126](https://www-ncbi-nlm-nih-gov.proxy.library.adelaide.edu.au/pubmed/?cmd=HistorySearch&querykey=7) |
| 8 | 5 AND 6 AND 7 | [142](https://www.ncbi.nlm.nih.gov/pubmed/?cmd=HistorySearch&querykey=24) |
| 9 | Filters: published in the last 10 years | 124 |

**Search strategy in PubMed**

Search ((((((((randomized controlled trial[pt] OR controlled clinical trial[pt] OR randomized[tiab] OR placebo[tiab] OR “clinical trials as topic”[majr] OR randomly[tiab] OR trial[ti]))) AND (("blood glucose self-monitoring"[mh] OR Blood Glucose Self-Monitoring[tiab] OR Blood Sugar Self-Monitoring[tiab] OR Home Blood Glucose Monitoring[tiab] OR Blood glucose monitoring system*[tiab]))) AND ((Continuous glucose monitoring system*[tiab] OR continuous glucose monitor*[tiab] OR continuous glucose sen*[tiab] OR continuous glucose device*[tiab] OR continuous blood sugar monitor*[tiab] OR continuous blood sugar sen*[tiab] OR continuous blood device*[tiab] OR continuous subcutaneous glucose monitor*[tiab] OR continuous subcutaneous glucose sen*[tiab] OR CGM*[tiab] OR real-time CGM*[tiab] OR rt-CGM*[tiab] OR flash glucose monitor*[tiab] OR FGM*[tiab] OR sensor-augmented insulin pump[tiab] OR SAP[tiab] OR iPro*[tiab] OR FreeStyle Libre*[tiab] OR HiBell*[tiab] OR Dexcom*[tiab] OR MiniMed*[tiab] OR Medtronic*[tiab] OR Guardian Connect CGM*[tiab] OR Senseonics Eversense*[tiab] OR GlucoTrack*[tiab]))) AND (("Diabetes mellitus, type 2"[mh] OR Type 2 diabetes[tiab] OR Ketosis resistant diabetes mellitus[tiab] OR Non insulin dependent diabetes[tiab] OR Stable diabetes mellitus[tiab] OR Diabetes mellitus[tiab] OR NIDDM[tiab] OR Maturity onset diabetes mellitus[tiab] OR MODY[tiab] OR Slow onset diabetes mellitus[tiab] OR Noninsulin-dependent diabetes mellitus[tiab] OR Noninsulin dependent diabetes mellitus[tiab] OR Maturity onset diabetes[tiab] OR Adult-Onset Diabetes Mellitus[tiab])))) AND English[Language]) AND Humans[Filter] Filters: published in the last 10 years
