# Supplementary material for: Comparing effects of continuous glucose monitoring systems (CGMs) and self-monitoring of blood glucose (SMBG) amongst adults with type 2 diabetes mellitus: a systematic review protocol
Source: Syst Rev. 2020 May 31;9:120. doi: 10.1186/s13643-020-01386-7 (PMC7262745; doi:10.1186/s13643-020-01386-7)
Supplement: Supplementary file 3 — Additional file 3: Logic grid. [file 13643_2020_1386_MOESM3_ESM.docx]

**Additional file 3: Logic Grid**

***Comparing effects of continuous glucose monitoring systems (CGMs) and self monitoring of blood glucose (SMBG) among adults with type 2 diabetes mellitus: Protocol for a systematic review***

**Limited to Humans and English**

**Search strategy below modified by RF (Librarian)**

| **Population**  **(Adults with type 2 diabetes)** | **Intervention**  **(All type of continuous glucose monitoring)** | **Control**  **(blood glucose self-monitoring or care )** | **Study** |
| --- | --- | --- | --- |
| **"Diabetes mellitus, type 2"[mh] OR**  Type 2 diabetes[tiab] OR  Ketosis resistant diabetes mellitus[tiab] OR  Non insulin dependent diabetes[tiab] OR  Stable diabetes mellitus[tiab] OR  Diabetes mellitus[tiab] OR  NIDDM[tiab] OR  Maturity onset diabetes mellitus[tiab] OR  MODY[tiab] OR  Slow onset diabetes mellitus[tiab] OR  Noninsulin-dependent diabetes mellitus[tiab] OR  Noninsulin dependent diabetes mellitus[tiab] OR  Maturity onset diabetes[tiab] OR  Adult-Onset Diabetes Mellitus[tiab] | **Continuous glucose monitoring system*[tiab] OR**  continuous glucose monitor*[tiab] OR  continuous glucose sen*[tiab] OR  continuous glucose device*[tiab] OR  continuous blood sugar monitor*[tiab] OR  continuous blood sugar sen*[tiab] OR  continuous blood device*[tiab] OR  continuous subcutaneous glucose monitor*[tiab] OR  continuous subcutaneous glucose sen*[tiab] OR  CGM*[tiab] OR  real-time CGM*[tiab] OR  rt-CGM*[tiab] OR  flash glucose monitor*[tiab] OR  FGM*[tiab] OR  sensor-augmented insulin pump[tiab] OR  SAP[tiab] OR  iPro*[tiab] OR  FreeStyle Libre*[tiab] OR  HiBell*[tiab] OR  Dexcom*[tiab] OR  MiniMed*[tiab] OR  Medtronic*[tiab] OR  Guardian Connect CGM*[tiab] OR  Senseonics Eversense*[tiab] OR  GlucoTrack*[tiab] | **"blood glucose self-monitoring"[mh] OR**  Blood Glucose Self-Monitoring[tiab] OR  Blood Sugar Self-Monitoring[tiab] OR  Home Blood Glucose Monitoring[tiab] OR  Blood glucose monitoring system*[tiab] | randomized controlled trial[pt] OR controlled clinical trial[pt] OR randomized[tiab] OR placebo[tiab] OR “clinical trials as topic”[majr] OR randomly[tiab] OR trial[ti] |

**Search strategy in PubMed**

((("Diabetes mellitus, type 2"[mh] OR Type 2 diabetes[tiab] OR Ketosis resistant diabetes mellitus[tiab] OR Non insulin dependent diabetes[tiab] OR Stable diabetes mellitus[tiab] OR Diabetes mellitus[tiab] OR NIDDM[tiab] OR Maturity onset diabetes mellitus[tiab] OR MODY[tiab] OR Slow onset diabetes mellitus[tiab] OR Noninsulin-dependent diabetes mellitus[tiab] OR Noninsulin dependent diabetes mellitus[tiab] OR Maturity onset diabetes[tiab] OR Adult-Onset Diabetes Mellitus[tiab])) AND ((Continuous glucose monitoring system*[tiab] OR continuous glucose monitor*[tiab] OR continuous glucose sen*[tiab] OR continuous glucose device*[tiab] OR continuous blood sugar monitor*[tiab] OR continuous blood sugar sen*[tiab] OR continuous blood device*[tiab] OR continuous subcutaneous glucose monitor*[tiab] OR continuous subcutaneous glucose sen*[tiab] OR CGM*[tiab] OR real-time CGM*[tiab] OR rt-CGM*[tiab] OR flash glucose monitor*[tiab] OR FGM*[tiab] OR sensor-augmented insulin pump[tiab] OR SAP[tiab] OR iPro*[tiab] OR FreeStyle Libre*[tiab] OR HiBell*[tiab] OR Dexcom*[tiab] OR MiniMed*[tiab] OR Medtronic*[tiab] OR Guardian Connect CGM*[tiab] OR Senseonics Eversense*[tiab] OR GlucoTrack*[tiab])) AND (("blood glucose self-monitoring"[mh] OR Blood Glucose Self-Monitoring[tiab] OR Blood Sugar Self-Monitoring[tiab] OR Home Blood Glucose Monitoring[tiab] OR Blood glucose monitoring system*[tiab])) AND ((randomized controlled trial[pt] OR controlled clinical trial[pt] OR randomized[tiab] OR placebo[tiab] OR “clinical trials as topic”[majr] OR randomly[tiab] OR trial[ti])

Filters: published in the last 10 years; Humans; English

Found results: 124
