# Supplementary material for: Comparing effects of continuous glucose monitoring systems (CGMs) and self-monitoring of blood glucose (SMBG) amongst adults with type 2 diabetes mellitus: a systematic review protocol
Source: Syst Rev. 2020 May 31;9:120. doi: 10.1186/s13643-020-01386-7 (PMC7262745; doi:10.1186/s13643-020-01386-7)
Supplement: Supplementary file 4 — Additional file 4: PRISMA-2009 flow diagram. [file 13643_2020_1386_MOESM4_ESM.doc]

**Additional file 4: PRISMA-2009 Flow Diagram**

**Screening**

**Included**

**Eligibility**

**Identification**

Additional records identified through other sources
(n = )

Eligible records after title and abstract screening
(n = )

Grey literature

(n = )

PubMed

(n = )

Cochrane Library

(n = )

Scopus

(n = )

EMBASE

(n = )

CINAHL

(n = )

PsycINFO (n = )

Records identified through database searching
(n = )

Records after duplicates removed
(n = )

Records excluded after reviewing title and abstract
(n = )

Records screened
(n = )

Full-text articles excluded

with reasons
(n = )

Studies included in quantitative synthesis

(meta-analysis)
(n = )
