# Supplementary material for: Comparing effects of continuous glucose monitoring systems (CGMs) and self-monitoring of blood glucose (SMBG) amongst adults with type 2 diabetes mellitus: a systematic review protocol
Source: Syst Rev. 2020 May 31;9:120. doi: 10.1186/s13643-020-01386-7 (PMC7262745; doi:10.1186/s13643-020-01386-7)
Supplement: Supplementary file 5 — Additional file 5: Data extraction form. [file 13643_2020_1386_MOESM5_ESM.docx]

**Additional file 5: Data Extraction Form**

***Comparing effects of continuous glucose monitoring systems (CGMs) and self monitoring of blood glucose (SMBG) among adults with type 2 diabetes mellitus: A systematic review protocol***

**Study ID: Data extractor: Date form completed (dd/mm/yy):**

| **Data to be extracted** | **Notes to reviewer** |
| --- | --- |
| First author: |  |
| Year of study: |  |
| Sample size (Sample size of T2D): |  |
| Title of study: | |

**1. General information**

| Publication type ⬜ Journal Article ⬜ Abstract ⬜ Conferences ⬜ Other (specify e.g. book chapter)_________ __________ | |
| --- | --- |
| Funding source of study: | Potential conflict of interest from funding? Y / N / unclear |
| Country of study: |  |
| Study duration (follow-up duration) | (⬜ ≥ 6 weeks ⬜ ＜ 6 weeks) |
| Type of participants | ⬜ Type 1 diabetes only ⬜ Type 2 diabetes only ⬜ Type 1&2 diabetes  ⬜ Other______ |
| Type of devices: | CGM (specify): _______ _______ (Real-time, Flash, Professional, Resorptive et al)  SMBG (specify): _________________________ Other (specify): __________________________ |

**2. Study eligibility**

| **Study Characteristics** |  | | Page/Figure |
| --- | --- | --- | --- |
| Type of study | ⬜ Randomised Controlled Trial (RCT)  ⬜ Cluster Randomised Controlled Trial (cluster RCT)  ⬜ Other (specify): ______________ | |  |
|  | *Does the study design meet the criteria for inclusion?*  Yes ⬜ No ⬜ 🡪Exclude Unclear ⬜ | |  |
| Participants  (Studies involving adults with type 2 diabetes using CGM systems for diabetes management) | Describe the participants included:  Age (years old): Mean ±SD  Gender: N _female_= N _male_=  Duration of diabetes: | |  |
|  | Are participants defined as a group having specific demographic, social or cultural characteristics? | Yes ⬜ No ⬜ Unclear ⬜  Details: |  |
|  | How is the geographic boundary defined? | Details:  Specific location (e.g. state / country): |  |
|  | *Do the participants meet one of the criteria for inclusion?*  a) adolescents (under 18 years of age) and elderly people (over 70)  b) other types of diabetes (i.e. gestational diabetes mellitus or idiopathic diabetes or type 1 diabetes). | Yes ⬜🡪Exclude No ⬜ Unclear ⬜ |  |
|  | *Do the participants meet the criteria for inclusion?* | Yes ⬜ No ⬜ 🡪Exclude Unclear ⬜ |  |
| Types of intervention | Strategies included in the intervention |  |  |
|  | Focus of the intervention |  |  |
|  | *Does the intervention meet the criteria for inclusion?* | Yes ⬜ No ⬜ 🡪Exclude Unclear ⬜ |  |
| Types of outcome measures | Outcomes: | Is HbA1c included as the outcomes?  Yes ⬜ No ⬜ Unclear ⬜ |  |
|  | *Do the outcome measures meet the criteria for inclusion?* | Yes ⬜ No ⬜ 🡪Exclude Unclear ⬜ |  |

**3. Summary of Assessment for Inclusion**

| Include in review ⬜ Exclude from review ⬜ | |
| --- | --- |
| Independently assessed, and then compared? Yes ⬜ No ⬜ | Differences resolved Yes ⬜ No ⬜ |
| Notes: | |
